# Implementation status of morbidity and mortality conferences in Austrian hospitals-A cross-sectional national survey study

**DOI:** 10.1371/journal.pone.0248692

**Published:** 2021-03-17

**Authors:** Alexandru-Cristian Tuca, Johanna Münch, David L. B. Schwappach, Andrea Borenich, Chiara Banfi, Selma Mautner, Magdalena Hoffmann, Christine Schwarz, Lars-Peter Kamolz, Gernot Brunner, Gerald Sendlhofer

**Affiliations:** 1 Research Unit for Safety in Health, c/o Division of Plastic, Aesthetic and Reconstructive Surgery, Department of Surgery, Medical University of Graz, Graz, Austria; 2 Swiss Patient Safety Foundation Zurich, Zürich, Switzerland; 3 Institute of Social and Preventive Medicine, University of Bern, Bern, Switzerland; 4 Institute for Medical Informatics, Statistics and Documentation, Medical University of Graz, Graz, Austria; 5 Executive Department for Quality and Risk Management, University Hospital Graz, Graz, Austria; 6 Austrian Society for Quality and Safety in Healthcare (ASQS), Graz, Austria; University Hospital Zurich, SWITZERLAND

## Abstract

**Introduction:**

Morbidity and mortality conferences (M&MCs) are an instrument for learning from past complications, unexpected follow-ups and deaths in hospitals and are important for improving patient safety. However, there are currently no quantitative data on the implementation of M&MCs in Austria. The aim of the study was to determine the status quo of the M&MCs in Austria.

**Materials and methods:**

A national cross-sectional study was conducted by means of a survey of 982 chief physicians of surgical disciplines, internal medicine, anesthesiology, intensive care, gynecology/obstetrics and pediatrics. The questionnaire focused on overall goals, structure and procedures of hospital M&MCs.

**Results:**

Of the 982 contacted chief physicians, 314 (32.0%) completed the survey. Almost two thirds of the respondents, i.e. 203 (64.7%), had already implemented M&MCs. Of the 111 chief physicians who had not yet introduced M&MCs, 62 (55.9%) were interested in introducing such conferences in the future. Of the 203 respondents that had implemented M&MCs, 100 stated that their M&MC could be improved. They reported issues with "shame and blame" culture, hierarchical structures, too little knowledge about the capability of M&MC and, in particular, time constraints. Overall, the participating chief physicians showed that they are striving to improve their existing M&MCs.

**Discussion/Conclusion:**

While we found a relatively high number of already implemented M&MCs we also identified a large heterogeneity in the format of the M&MCs. A highly structured M&MC including guidelines, checklists or templates does not only considerably improve its outcome but can also alleviate the main limiting factor which is the lack of time.

## Introduction

“To Err is Human”–a report, published by the Institute of Medicine in 1999 showed that errors and mistakes with consecutive minor and major complications happen regularly in clinical routine [1]. While it is not possible to change the human nature to err, it is possible to learn from errors and mistakes and prevent future occurrences. One powerful clinical tool for promoting patient safety are morbidity and mortality conferences (M&MCs). An M&MC is a system-based tool that helps to discuss and understand medical errors or complications and to make them less likely to occur again in the same way [2].

Canada, the USA and UK have a long tradition of M&MCs compared to German-speaking countries. At the beginning of the 20^th^ century, the American surgeon Ernest Armory Codman mentioned the need for a conference to compare the results of different treatments and to improve the overall quality of patient care by using transparent results [3]. In the USA, follow-up of adverse events, near misses, and unsafe conditions is now a mandatory requirement for internal and surgical wards to obtain accreditation as a teaching hospital by the Accreditation Counsel for Graduate Medicine Education [4]. Since 2010, M&MCs are also mandatory in France for oncological, surgical, anesthesiologic and intensive care units [5].

Based on years of intensive experience in North America and the UK, existing M&MCs have been reformed. Furthermore, standards and guidelines have been developed helping to increase effectiveness and quality [5,6]. In German-speaking countries M&MCs are more or less poorly implemented, but several guidelines have been published by the German Medical Association and the Swiss Patient Safety Foundation that describe the importance of a clear distinction between M&MCs and ordinary case conferences [7–9]. The Foundations also state that M&MCs should not be limited to teaching students, but should also be used for education, training, and continuing education of doctors, as well as for system improvement. The result of M&MCs should not only increase the knowledge of the participants, but also enhance structural and process benefits. The guidelines also mention that there are no internationally consistent guidelines and that different approaches can be found in the literature [8,9]. Although M&MCs are not as frequently reported in German-speaking countries some surveys on existing conferences have already been published. Recently, two studies surveyed chief physicians and asked how M&MCs are implemented in Switzerland and Germany [7,10,11]. Results showed that respondents were satisfied and perceived M&MCs as an efficient tool. Furthermore, chief physicians expressed the need for structural und procedural improvement and standardization.

To our knowledge, no information on the implementation of M&MCs is available in Austria so far. This information led us to the hypothesis, that M&MCs are only conducted rarely in Austrian hospitals and that no standardized guidelines are used to lead through the process, which would show in rather unsatisfying results of the conferences. The aim of this study was to use the already validated and successfully utilized questionnaire from the Swiss Patient Safety Foundation to assess the current situation in Austria by contacting chief physicians in Austrian hospitals [7]. Moreover, we investigated whether the implementation of M&MCs was associated with hospital characteristics such as, for example, the number of beds, the legal structure, the type of medical departments or the number of beds per department.

## Materials and methods

A cross-sectional hybrid survey (sent online and hard copy by postal services) was conducted in October 2018. The survey was approved by the Ethics Committee, Medical University of Graz (30–416 ex 17/18). The participants were informed at the very beginning of the survey in written form, that it is voluntary to take part and that stepping back will be possible at all times. Additionally, they were informed that all data will be pseudonymised. The Ethics Committee agreed to this consent information process.

### Survey instrument

For the present study a validated and successfully used questionnaire of the Swiss Patient Safety Foundation was used with its permission [7,11]. The questionnaire is based on assessments and internationally established guidelines, such as the Ottawa M&M Model and the recommendations of the German Medical Association [8,12]. It consists of three sections: 1) Whether M&MCs are currently implemented at the clinic or department. In case M&MCs were not implemented, physicians were asked whether they would be interested in introducing M&MCs to their staff. 2) Overall goals, structure and process elements of M&MCs. 3) Satisfaction and perceived effectiveness of M&MCs. Answer options were binary or categorical and some questions provided free-text fields. In total, the questionnaire consisted of 44 items.

### Sample and procedures

In this study, chief physicians were contacted either by email or by letter and asked to participate in the survey. The questionnaire was sent to every chief physician in Austria, no departments or hospitals were excluded. If some specialties are not represented in the study, it is because they chose not to participate or did not indicate their specialty.

The goal was, to include all Austrian hospitals in the survey, hence the sample size. The email addresses were determined based on the list of Austrian hospitals of the Federal Ministry of Health or, in case of not finding an email address, the department was contacted by postal letter. Thus, the survey was conducted as a hybrid survey and used the questionnaire of the Swiss Patient Safety Foundation. The duration of the survey was 4 weeks and a total of three reminder emails were sent to those who were invited by email, in case there was no response. The online survey was made available via the EVASYS survey system (EvaSys V7.1, Electric Paper Evaluationssysteme GmbH, Lüneburg, Germany). Questionnaires returned by post were scanned. All questionnaires were evaluated with the EVASYS survey system.

### Data analysis

Analyses were performed using R (version 3.6.1). Data were descriptively summarized as absolute and relative frequencies. Differences between groups were determined by the Chi-squared test for categorical data. In case the assumptions of the chi-squared test were not met (expected counts < 5 and/or observed cells with zero observations) Fisher tests were used. A p value <0.05 was considered significant.

## Results

### Sample

Of the 982 contacted chief physicians, 314 (32.0%) completed the survey. 203 respondents (64.7%) indicated that M&MCs had been implemented in their units. Surgical and internal disciplines showed the highest rates of survey participations with 32.8% and 20.7% respectively, whereas obstetrics/gynecology and pediatrics had the lowest rates with 7.0% and 3.2%, respectively. 111 clinicians (35.4%) reported that they have not implemented M&MCs, but 62 of them (55.9%) were interested to do so and 37 were “maybe interested” (33.3%). Twelve of these 111 clinicians (10.8%) had no interest in organizing M&MCs in the near future. Hospital characteristics are presented in Table 1.

**Table 1:** Demographics.

	M&M currently implemented (N = 203)	M&M currently not implemented (N = 111)	Total (N = 314)	p value
**Gender**				0.871^1^
N-Miss	4	6	10	
Male	175 (87.9%)	93 (88.6%)	268 (88.2%)	
Female	24 (12.1%)	12 (11.4%)	36 (11.8%)	
**Hospital categories**				0.003^2^
N-Miss	4	5	9	
University hospital	44 (22.1%)	24 (22.6%)	68 (22.3%)	
General hospital with more than 500 beds	65 (32.7%)	22 (20.8%)	87 (28.5%)	
General hospital with 125–499 beds	84 (42.2%)	45 (42.5%)	129 (42.3%)	
General hospital with less than 124 beds	4 (2.0%)	12 (11.3%)	16 (5.2%)	
Special clinics	2 (1.0%)	3 (2.8%)	5 (1.6%)	
**Legal structure**				0.002^2^
N-Miss	6	1	7	
Public hospital	178 (90.4%)	107 (97.3%)	285 (92.8%)	
Private hospital	0 (0.0%)	1 (0.9%)	1 (0.3%)	
Religious hospital	18 (9.1%)	1 (0.9%)	19 (6.2%)	
Other hospital	1 (0.5%)	1 (0.9%)	2 (0.7%)	
**Medical discipliness**				0.002^2^
Surgery	79 (38.9%)	24 (21.6%)	103 (32.8%)	
Internal medicine	37 (18.2%)	28 (25.2%)	65 (20.7%)	
Anaesthesiology & intensive care	28 (13.8%)	10 (9.0%)	38 (12.1%)	
Obstetrics/gynaecology	16 (7.9%)	6 (5.4%)	22 (7.0%)	
Pediatrics	7 (3.4%)	3 (2.7%)	10 (3.2%)	
not reported	36 (17.7%)	40 (36.0%)	76 (24.2%)	
**Number of beds in department**				0.708^1^
N-Miss	14	12	26	
Up to 20	22 (11.6%)	15 (15.2%)	37 (12.8%)	
21–40	50 (26.5%)	28 (28.3%)	78 (27.1%)	
41–60	40 (21.2%)	15 (15.2%)	55 (19.1%)	
61–80	29 (15.3%)	12 (12.1%)	41 (14.2%)	
Over 80	32 (16.9%)	18 (18.2%)	50 (17.4%)	
Not applicable	16 (8.5%)	11 (11.1%)	(9.4%)	

*Note*. N-Miss indicates the number of missing values.

^1^ Pearson’s Chi-squared test.

^2^ Fisher test.

### M&MCs goals

**Fig 1:**
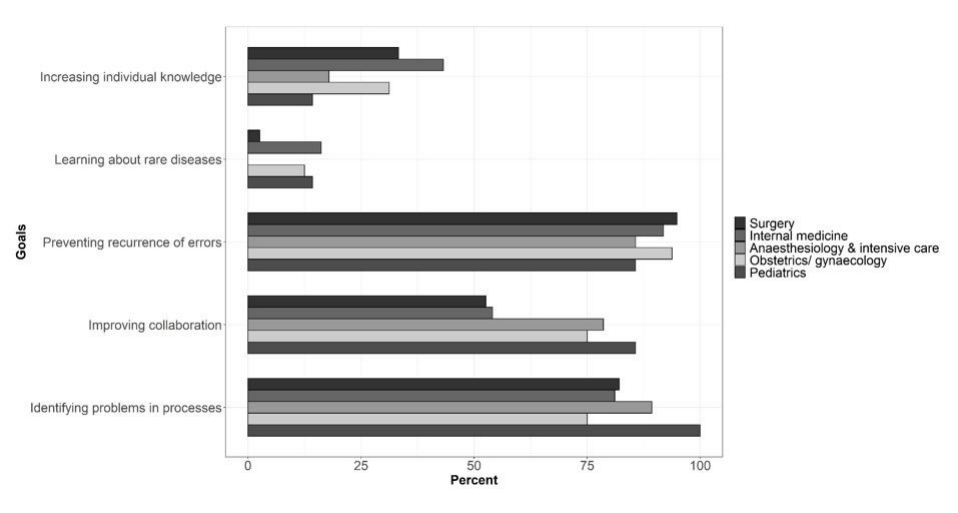
Goals presented separately for each medical discipline.

Respondents had to choose three out of five goals which they wanted to improve by using M&MCs. The most commonly chosen goals relate to organizational improvement: ‘Preventing recurrence of errors’ (91.6%), ‘Identifying problems in the processes’ (83.2%) and ‘Improving collaboration between professionals and departments’ (60.4%). Goals about individual learning were indicated less frequently: ‘Increasing individual knowledge’ (34.7%) and ‘Learning about rare diseases’ (6.9%) Several differences between medical disciplines and their chosen goals were found (see Fig 1). The overall goal ‘Preventing recurrence of errors’ showed a frequency of more than 90% for representatives of surgery (94.9%), internal medicine (91.9%) and obstetrics/gynecology (93.8%). ‘Improving collaboration’ was essential for representatives of intensive care and pediatrics (78.6% and 85.7% respectively). But less important compared to representatives from surgery (52.6%) and internal medicine (54.1%).

### M&MCs characteristics

As reported in Table 2, survey respondents reported that M&MCs were held irregularly (39.6%), quarterly (36.0%), monthly (20.3%) and weekly (4.1%). Most M&MCs were reported to last approximately 60 minutes (26.1%) or longer (35.0%). The remaining respondents reported to discuss their cases in 30 to 45 minutes (19.2%) or less than 30 minutes (19.7%). In these M&MCs, mostly one or two cases (46.0% and 26.7% respectively) were discussed, the remaining 27.3% of respondents reported that three or more cases are discussed. Open discussion for each case lasting more than 20 minutes was reported in 26.6%, whereas the remaining respondents reported briefer durations. Between clinical disciplines, significant differences were found concerning how many cases are discussed (p = 0.023) and how much time is used for open discussions (p = 0.011).

**Table 2:** Characteristics for responders that currently implement M&MCs (N = 203).

	Surgery (N = 79)	Internal medicine (N = 37)	Anaesthesiology & intensive care (N = 28)	Obstetrics/gynaecology (N = 16)	Pediatrics (N = 7)	Not reported (N = 36)	Total (N = 203)	p value
**Frequency**								0.057^1^
N-Miss	1	2	0	0	1	2	6	
Weekly/fortnightly	4 (5.1%)	1 (2.9%)	0 (0.0%)	0 (0.0%)	0 (0.0%)	3 (8.8%)	8 (4.1%)	
Monthly	24 (30.8%)	5 (14.3%)	4 (14.3%)	2 (12.5%)	0 (0.0%)	5 (14.7%)	40 (20.3%)	
Quarterly	24 (30.8%)	13 (37.1%)	7 (25.0%)	10 (62.5%)	1 (16.7%)	16 (47.1%)	71 (36.0%)	
< = quarterly/irregular	26 (33.3%)	16 (45.7%)	17 (60.7%)	4 (25.0%)	5 (83.3%)	10 (29.4%)	78 (39.6%)	
**Duration**								0.328^1^
N-Miss	0	0	0	0	0	0	0	
< = 30 min	16 (20.3%)	5 (13.5%)	6 (21.4%)	4 (25.0%)	3 (42.9%)	6 (16.7%)	40 (19.7%)	
31–45 min	18 (22.8%)	6 (16.2%)	4 (14.3%)	3 (18.8%)	0 (0.0%)	8 (22.2%)	39 (19.2%)	
46–60 min	16 (20.3%)	16 (43.2%)	6 (21.4%)	1 (6.2%)	2 (28.6%)	12 (33.3%)	53 (26.1%)	
>60 min	29 (36.7%)	10 (27.0%)	12 (42.9%)	8 (50.0%)	2 (28.6%)	10 (27.8%)	71 (35.0%)	
**Number of attendees**								0.799^1^
N-Miss	3	4	3	1	1	7	19	
2–10	13 (17.1%)	9 (27.3%)	6 (24.0%)	3 (20.0%)	1 (16.7%)	7 (24.1%)	39 (21.2%)	
11–20	31 (40.8%)	10 (30.3%)	9 (36.0%)	4 (26.7%)	2 (33.3%)	12 (41.4%)	68 (37.0%)	
21–30	9 (11.8%)	7 (21.2%)	3 (12.0%)	1 (6.7%)	0 (0.0%)	1 (3.4%)	21 (11.4%)	
>30	23 (30.3%)	7 (21.2%)	7 (28.0%)	7 (46.7%)	3 (50.0%)	9 (31.0%)	56 (30.4%)	
**Estimated participation rate of invited staff**								0.865^1^
N-Miss	1	2	3	1	2	5	14	
1–25%	4 (5.1%)	1 (2.9%)	3 (12.0%)	1 (6.7%)	0 (0.0%)	4 (12.9%)	13 (6.9%)	
26–50%	11 (14.1%)	8 (22.9%)	4 (16.0%)	3 (20.0%)	0 (0.0%)	5 (16.1%)	31 (16.4%)	
51–75%	26 (33.3%)	15 (42.9%)	7 (28.0%)	4 (26.7%)	2 (40.0%)	10 (32.3%)	64 (33.9%)	
76–100%	37 (47.4%)	11 (31.4%)	11 (44.0%)	7 (46.7%)	3 (60.0%)	12 (38.7%)	81 (42.9%)	
**No. of cases per M&MC**								0.023^1^
N-Miss	5	1	4	0	1	5	16	
1 case	28 (37.8%)	16 (44.4%)	18 (75.0%)	6 (37.5%)	5 (83.3%)	13 (41.9%)	86 (46.0%)	
2 cases	19 (25.7%)	15 (41.7%)	4 (16.7%)	4 (25.0%)	0 (0.0%)	8 (25.8%)	50 (26.7%)	
3 cases	10 (13.5%)	3 (8.3%)	2 (8.3%)	3 (18.8%)	1 (16.7%)	7 (22.6%)	26 (13.9%)	
> = 4 cases	17(23.0%)	2 (5.6%)	0 (0.0%)	3 (18.8%)	0 (0.0%)	3 (9.7%)	25 (13.4%)	
**Time per case for open discussion**								0.011^1^
N-Miss	7	1	2	1	0	4	15	
< = 10 min	15 (20.8%)	3 (8.3%)	2 (7.7%)	2 (13.3%)	0 (0.0%)	8 (25.0%)	30 (16.0%)	
11–15 min	21 (29.2%)	11 (30.6%)	3 (11.5%)	1 (6.7%)	2 (28.6%)	12 (37.5%)	50 (26.6%)	
16–20 min	16 (22.2%)	16 (44.4%)	11 (42.3%)	9 (60.0%)	1 (14.3%)	5 (15.6%)	58 (30.9%)	
>20 min	20 (27.8%)	6 (16.7%)	10 (38.5%)	3 (20.0%)	4 (57.1%)	7 (21.9%)	50 (26.6%)	
**Presenter**								0.215^1^
N-Miss	11	6	4	1	2	11	35	
Chief physician	10 (14.7%)	2 (6.5%)	1 (4.2%)	3 (20.0%)	1 (20.0%)	2 (8.0%)	19 (11.3%)	
Senior physician	38 (55.9%)	20 (64.5%)	20 (83.3%)	5 (33.3%)	2 (40.0%)	14 (56.0%)	99 (58.9%)	
Resident	15 (22.1%)	7 (22.6%)	2 (8.3%)	7 (46.7%)	2 (40.0%)	6 (24.0%)	39 (23.2%)	
Other	5 (7.4%)	2 (6.5%)	1 (4.2%)	0 (0.0%)	0 (0.0%)	3 (12.0%)	11 (6.5%)	
**Role allocation**								0.248^1^
N-Miss	2	0	0	0	0	2	4	
1 person chairs, moderates and presents (all in one)	16 (20.8%)	3 (8.1%)	1 (3.6%)	3 (18.8%)	0 (0.0%)	6 (17.6%)	29 (14.6%)	
1 person chairs and moderates & 1 or more others persons present	45 (58.4%)	29 (78.4%)	23 (82.1%)	12 (75.0%)	6 (85.7%)	18 (52.9%)	133 (66.8%)	
1 person chairs & 1 person moderates & 1 or several persons present	8 (10.4%)	2 (5.4%)	3 (10.7%)	1 (6.2%)	0 (0.0%)	7 (20.6%)	21 (10.6%)	
1 person chairs & 1 or several other persons present	8 (10.4%)	3 (8.1%)	1 (3.6%)	0 (0.0%)	1 (14.3%)	3 (8.8%)	16 (8.0%)	
**Is the moderator trained**								0.563^1^
N-Miss	5	2	1	1	1	9	19	
Yes	27 (36.5%)	8 (22.9%)	9 (33.3%)	5 (33.3%)	3 (50.0%)	5 (18.5%)	57 (31.0%)	
Partially	31 (41.9%)	15 (42.9%)	13 (48.1%)	6 (40.0%)	1 (16.7%)	16 (59.3%)	82 (44.6%)	
No	16 (21.6%)	12 (34.3%)	5 (18.5%)	4 (26.7%)	2 (33.3%)	6 (22.2%)	45 (24.5%)	
**Types of improvement measures**								0.528^1^
N-Miss	11	4	7	3	0	9	34	
Individual	7 (10.3%)	3 (9.1%)	3 (14.3%)	0 (0.0%)	0 (0.0%)	0 (0.0%)	13 (7.7%)	
Local	32 (47.1%)	18 (54.5%)	6 (28.6%)	6 (46.2%)	4 (57.1%)	14 (51.9%)	80 (47.3%)	
Systemic	29 (42.6%)	12 (36.4%)	12 (57.1%)	7 (53.8%)	3 (42.9%)	13 (48.1%)	(45.0%)	

*Note*. Missing values related to medical disciplines are listed in a separate column named “not reported”. N-Miss indicates the number of missing values for the other variables.

^1^ Fisher test.

Concerning task allocation during M&MCs, the most common model was using one chair person who also moderates, and one or more other persons who present (66.8%). Only 8.0% report to have no moderator.

Most reported improvements derived through M&MCs include ‘local measures that optimize a (sub-) process in one area and locally’ (47.3%) and ‘systemic measures that optimize a (partial) process for the entire hospital and have a global or systemic effect’ (45.0%). ‘Individual measures aimed at changing the behavior of individual employees’ are only rarely derived (7.7%).

Participants at M&MCs with a medical degree consist of (multiple selection possible): chief physicians (95.6%), senior physicians (97.5%), assistant physicians (94.1%), nurses (48.3%) and midwives (11.8%). Other participants of M&MCs are students, quality-management professionals and professionals from other disciplines.

The most common cases presented in M&MCs were ‘complications’ (68.3%), ‘unexpected mortality’ (47.5%) and ‘cases selected based on problems in cooperation’ (36.6%). Cases rarely presented in M&MCs were ‘cases with little conflict potential among the involved parties’ (0%), ‘rare diseases’ (6.4%) and ‘critical events with patient harm’ (17.3%).

### Procedural features of M&MCs

Detailed procedural features of M&MCs can be found in Table 2. The questionnaire included options whether the facilitator was trained, partially trained or not trained to perform this task during M&MCs. This was the case for 31.0%, 44.6% and 24.5% of respondents respectively.

### Satisfaction, perceived effectiveness and improvement potential

Regarding satisfaction with the current M&MCs at their units, 70.8% of respondents were ‘satisfied’ and 13.0% were ‘very satisfied’. The effectiveness of M&MCs was also rated positively with ‘effective’ (54.5%) and ‘very effective’ (38.0%). Only one respondent (0.5%) stated that M&MCs were non-effective. Potential for improvement of the current M&MCs was seen in 50.5%, whereas 38.9% stated no need for improvement.

### Factors limiting the effectiveness of M&MCs

The most common factors that hindered the effectiveness of M&MCs were (multiple selection possible): lack of time (72.6%), insufficient willingness to participate (19.9%), lack of methodological competences (12.4%), "shame and blame" culture (13.9%), absence of follow-up investigations (12.4%), hierarchical structures that prevent open discussions (5.5%), and too little knowledge about the potential of M&MC (4.0%).

## Discussion

The present study is the first one to identify and evaluate the current implementation of M&MCs in Austria by using an already validated and successfully utilized questionnaire from the Swiss Patient Safety Foundation. Overall, the response rate is considered high compared to similar studies. Participation rate was highest for the clinical disciplines of surgery and internal medicine (32.8% and 20.7% respectively). The present study shows that there is already a broad acceptance of M&MCs among Austrian chief physicians. 64.7% have already implemented M&MCs. Of the remaining 35.4%, 55.9% were interested in introducing M&MCs in the future. Nevertheless, one third of the participants thought that there is still potential for improvement in their current M&MCs.

In general, M&MCs are a useful tool to learn from past complications, unexpected follow-ups and deaths. These should be strictly distinguished from the Clinical Incident Reporting System (CIRS), which mainly deals with mistakes and “near-misses” that can affect patient safety [13]. M&MCs have been identified too as a tool to promote and engage in quality improvement and patient safety by reflecting, analyzing and discussing these events in a structured way [14–16]. In order to hold productive M&MCs, existing guidelines are listing several key points that are essential, e.g. clinic management has to support M&MCs, a facilitator is essential, conferences have to be held regularly and in a highly structured format, conferences should be attended by different disciplines and the setting should encourage open discussion without any blaming [17,18].

In the context of the present study, we were able to identify specific needs for improvement e.g. regarding the roles of active participants. By far the most frequently used method in Austria (66.8%), includes one person who leads and moderates the conference and a second person who presents. In the second highest ranked model (14.6%), one person is responsible for all three of these tasks. Both models are insufficient according to the guidelines which recommend one person for each of the following tasks: coordination, leadership, moderation and presentation [19]. In Austrian hospitals this kind of task allocation was found in only 10.6% of respondents. The German guideline even explicitly states that the tasks of leading and facilitating the conference should not be done by the same person since the facilitator should be a neutral person and should strive to ensure that the values of the M&MCs (no blaming, open atmosphere etc.) are adhered to. The chairperson (lead) of the meeting is an internal expert of the ward, if possible in a leading position and finally responsible for the discussed case. Since the chairperson is directly involved in the presented case, a neutral position is rather difficult to achieve. Therefore, a clear separation of these tasks will lead to better outcomes of the M&MC. A neutral facilitator is considered particularly important and should receive training for this task. In our study, 31.0% of the facilitators are currently trained. Both German and Swiss guidelines point out that trained facilitators are gold standard for M&MCs and are also essential to create an open, non-hierarchical atmosphere.

There is always room for improvement with regard to the regularity of M&MCs. While the German Medical Association recommends at least one conference per quarter, the Swiss Patient Safety Foundation believes that monthly conferences should be the minimum. In view of these recommendations, the majority of the Austrian departments studied already meet this criterion. But with 39.6% of conferences held less frequently there is still potential for improvement. The evaluation of the willingness to improve the regularity was not part of the present study but we think it depends on different factors like the size of the hospitals, the complexity of existing cases and of course the resources to perform such conferences more often.

A further core element of M&MCs is their interdisciplinary nature. In addition to physicians, other healthcare professionals who were involved in the case or work in the respective departments should also participate. In Austria, the nursing staff is currently involved in less than 50% of the conferences. The inclusion of risk and quality management (RM/QM) staff is considered important in the guidelines, as they often have a better overview and are therefore better able to identify problem patterns. In our survey, 30.2% of the respondents stated that employees from RM/QM are present at the conferences. The management employees are even less frequently involved (6.8%). Affiliated physicians and specialists from outside the hospital are invited to the conferences by less than 3%. According to the guidelines, mandatory participation in the conferences and the final number of participants should be decided in the department and is left to the discretion of those responsible.

Between the disciplines we detected differences especially between internal medicine and surgery, which were the specialties with most respondents. We could see a longer duration per case and also a longer overall duration of M&MCs in surgery departments. Further, the frequency of M&MCs in surgery departments was more often than in internal medicine departments, which is consistent with literature [7,20].

An important aspect of M&MCs is a highly structured format for the conferences. Due to the often-large number of participants at M&MCs, a structured format is essential tool to achieve transparency, understanding and maximum performance. Furthermore, it reduces the time for preparation and ensures clear expectations and outcomes of the conferences. Clear and objective criteria for case selection are of major importance to avoid perceptions that the M&MCs are misused to penalize certain members of staff. Outcomes of discussing the presented cases include immediate measures and long-term lessons that can prevent errors in the future. A regular follow-up during subsequent M&MCs and information regarding the implementation of already decided measures, as described in the guidelines, will increase motivation to participate.

Guidelines also recommend that concise and anonymous meeting minutes should be taken as a written record of each conference to enable those who are absent to inform themselves afterwards and to forward the results to the RM/QM. In our study a great majority indicated to write minutes, but only half did it anonymously. In principle, anonymization applies both to patient data in the sense of medical confidentiality and to employees as protection against public blame for their mistakes. The publication of names can be an obstacle for future cases and reduce compliance and employee confidence in M&MCs. Beside improving quality, M&MCs also serve as an educational instrument for students, residents and other medical staff [20,21].

## Conclusion

Of course, M&MCs are a double-edged sword. If there is a lack of recognition and rules for holding these conferences, the results can be very counterproductive. On the other hand, if the format is highly structured and guidelines are followed, the positive impact on patient safety could be much greater than the investment of time and money. Our survey shows that Austrian hospitals are already implementing M&MCs but there is still room for improvement. By performing similar surveys in the future, progress in the implementation of M&MCs in Austria can be monitored and can contribute to continuous improvement.

### Limitations

The self-reporting nature of the data in this publication is one of the main limitations. Although the response rate was considered high, standard limitations associated with a response rate below 100% must be taken into account, such as over- and under-representation of certain disciplines.

## Supporting information

S1 File Participating hospitals and institutions. (PDF)
