# Supplementary material for: Implementation status of morbidity and mortality conferences in Austrian hospitals-A cross-sectional national survey study
Source: PLoS One. 2021 Mar 17;16(3):e0248692. doi: 10.1371/journal.pone.0248692 (PMC7968694; doi:10.1371/journal.pone.0248692)
Supplement: S1 File — (PDF) [file pone.0248692.s001.pdf]

S1 File. Participating hospitals and institutions

1) Rehabilitationszentrum für Herz-Kreislauferkrankungen Bad Tatzmannsdorf
2) Krankenhaus der Barmherzigen Brüder Eisenstadt
3) Landeskrankenhaus Güssing
4) Ladislaus Batthyany-Strattmann Krankenhaus Kittsee
5) Landeskrankenhaus Oberpullendorf
6) Landeskrankenhaus Oberwart
7) Orthopädisches Klinikum SKA Zicksee
8) Heilpädagogisches Zentrum Rust
9) BVAEB Therapiezentrum Rosalienhof
10) Sonderkrankenanstalt Onkologische Rehabilitation - Der Sonnberghof
11) Sonderkrankenanstalt für neurologische Rehabilitation Kittsee
12) Krankenhaus des Deutschen Ordens Friesach GmbH
13) Gailtal-Klinik Hermagor
14) Klinikum - Klagenfurt am Wörthersee (LKH Klagenfurt)
15) Krankenhaus der Elisabethinen Klagenfurt GmbH
16) Unfallkrankenhaus Klagenfurt
17) Abteilung für chronische Kranke im Klinikum Klagenfurt am Wörthersee
18) Privatklinik Maria Hilf Klagenfurt
19) Landeskrankenhaus Laas
20) Krankenhaus der Barmherzigen Brüder St. Veit/Glan
21) Krankenhaus Spittal/Drau
22) Landeskrankenhaus Villach
23) Abteilung für chronisch Kranke im Landeskrankenhaus Villach
24) Krankenhaus Waiern
25) Landeskrankenhaus Wolfsberg
26) Abteilung für chronische Kranke und Zentrum für Lymphologie im Landeskrankenhaus Wolfsberg
27) Sonderkrankenanstalt de La Tour
28) Privatklinik Villach
29) Interdisziplinäre Sonderkrankenanstalt für Rehabilitation
30) Sonderkrankenanstalt Die Schrothkur Obervellach
31) Abteilung für chronisch Kranke im Landeskrankenhaus Laas
32) Sonderkrankenanstalt für Orthopädie und orthopädische Chirurgie Warmbad-Villach
33) Rehabilitationsklinik für seelische Gesundheit und Prävention
34) Sonderkrankenanstalt für medizinische Rehabilitation Thermenhof
35) KABEG Gailtalklinik
36) Sonderkrankenanstalt im "OptimaMed Gesundheitsresort Agathenhof"
37) Sonderkrankenhaus Sarepta-Spezialkliinik für Essstörungen
38) Landesklinikum Amstetten
39) Klinikum am Kurpark Baden für Orthopädie und Rheumatologie
40) Sonderkrankenanstalt Bad Schönau
41) SKA-RZ-Pensionsversicherungsanstalt Felbring im Schneebergland
42) Landesklinikum Hochegg
43) Landesklinikum Hainburg
44) Rehabilitationszentrum Hochegg
45) Landesklinikum Hollabrunn
46) Landesklinikum Klosterneuburg
47) Universitätsklinikum Krems
48) Rehabilitationszentrum für Bewegungsstörungen und rheumatische Erkrankungen
49) Landesklinikum Mauer
50) Landesklinikum Melk
51) Landesklinikum Mistelbach-Gänserndorf

52) Landesklinikum Neunkirchen
53) Sonderkrankenanstalt Gesundheitsresort Raxblick
54) Landesklinikum Scheibbs
55) Sonderkrankenanstalt der Justizanstalt Wien-Josefstadt (Außenstelle Wilhelmshöhe)
56) Landesklinikum Waidhofen/Ybbs
57) Landesklinikum Wiener Neustadt
58) Therapiezentrum Ybbs
59) Österreichische Gesundheitskasse - Mein Peterhof Baden
60) Klinikum Malcherhof Baden
61) BVAEB Gesundheitseinrichtung Breitenstein
62) Sonderkrankenanstalt für Herz- und Kreislauferkrankungen Groß Gerungs
63) Rehabilitationszentrum 'Weißer Hof' Klosterneuburg
64) Sonderkrankenanstalt Moorheilbad Harbach
65) Rehabilitationszentrum Alland
66) Klinik Pirawarth, Kur- und Rehabilitationszentrum
67) BVAEB Therapiezentrum Buchenberg
68) Landesklinikum Horn-Allentsteig
69) Landesklinikum Korneuburg-Stockerau
70) Universitätsklinikum Tulln
71) Landesklinikum Baden-Mödling-Hinterbrühl
72) Sonderkrankenanstalt Marienhof
73) Universitätsklinikum St. Pölten-Lilienfeld
74) Landesklinikum Zwettl-Gmünd-Waidhofen/Thaya
75) Psychosomatisches Zentrum Eggenburg - Rehabilitationsklinik Gars am Kamp
76) Sonderkrankenanstalt Ottenschlag
77) BVAEB Rehabilitationszentrum Engelsbad
78) Waldsanatorium Perchtoldsdorf
79) Sonderkrankenanstalt für Psychiatrie Krems-Hollenburg
80) Psychosomatisches Zentrum Eggenburg - Rehabilitationsklinik Gars am Kamp (gehört zu K384)
81) Sonderkrankenanstalt für Remobilisation/Nachsorge und onkologische Rehabilitation
82) kokon Bad Erlach, Reha für junge Menschen
83) Sonderkrankenanstalt für stationäre psychiatrische Rehabilitation
84) Krankenhaus "St. Josef" Braunau am Inn
85) Klinikum Freistadt
86) Sonderkrankenanstalt für neurologische und pneumologische Rehabilitation
87) Sanitätsanstalt Hörsching des Militärkommandos Oberösterreich
88) Krankenhaus der Barmherzigen Brüder Linz
89) Krankenhaus der Barmherzigen Schwestern vom Hl. Vinzenz von Paul Linz
90) Krankenhaus der Elisabethinen Linz
91) Unfallkrankenhaus Linz
92) Klinik Diakonissen Linz
93) Krankenhaus der Barmherzigen Schwestern vom Hl. Vinzenz von Paul Ried
94) Klinikum Schärding
95) Sonderkrankenanstalt Sierning
96) Klinikum Wels-Grieskirchen
97) Sonderkrankenanstalt zur Behandlung unspezifischer Atemwegserkrankungen, Erkrankungen des rheumatischen Formenkreises sowie für postoperative Rehabilitation
98) Klinikum Bad Hall für Herz- Kreislauf- und neurologische Rehabilitation
99) Sonderkrankenanstalt - Rehabilitationszentrum Bad Schallerbach (SKA - RZ Bad Schallerbach)
100) Klinikum Rohrbach
101) Rehabilitationszentrum Bad Ischl-Lindau
102) Rehabilitationszentrum St. Georgen

103) Sonderkrankenanstalt für Kinder- und Jugendneuropsychiatrie
104) HerzReha Herz Kreislaufzentrum Bad Ischl
105) Sonderkrankenanstalt für Herz- und Kreislauferkrankungen, Stoffwechselerkrankungen sowie Atemwegserkrankungen – BVAEB Rehabilitationszentrum Austria
106) Sonderkrankenanstalt Aspach
107) Sonderkrankenanstalt für neurologische und orth. Rehabilitation und Kinderrehabilitation
108) Sonderkrankenanstalt für Krankheiten des Stoffwechselsystems und des Verdauungsapparates
109) Sonderkrankenanstalt für neurologische Rehabilitation Altmünster
110) Klinikum Schallerbacherhof für orthopädische Rehabilitation
111) Pyhrn-Eisenwurzen Klinikum Kirchdorf Steyr
112) Linz Kepler Universitätsklinikum
113) Salzkammergut-Klinikum
114) Sonderkrankenanstalt Rehabilitationszentrum für psychische Rehabilitation in Bad Hall-BVAEB Therapiezentrum Justuspark
115) Rehabilitationszentrum Vortuna Bad Leonfelden
116) kokon Rohrbach-Berg Reha für junge Menschen
117) Rehabilitationszentrum Bad Hofgastein
118) Landesklinik Hallein
119) Aö Krankenhaus Oberndorf
120) Krankenanstalt Obertauern Dr. Aufmesser
121) Sanatorium Oberthurnhof St. Jakob am Thurn
122) Krankenanstalt Radstadt Dr. Aufmesser
123) Landeskrankenhaus Salzburg - Universitätsklinikum der PMU
124) Unfallkrankenhaus Salzburg
125) Privatklinik Wehrle - Diakonissen, Standort Aigen
126) Krankenhaus der Barmherzigen Brüder Salzburg
127) Christian-Doppler-Klinik Salzburg - Universitätsklinikum der PMU
128) Landesklinik St. Veit
129) Kardinal Schwarzenberg Klinikum
130) Landesklinik Tamsweg
131) A.ö. Tauernklinikum Standort Zell am See und Standort Mittersill
132) Klinikum Bad Gastein für Orthopädie und Rheumatologie
133) Suchthilfe Klinik Salzburg
134) Rehabilitationszentrum Saalfelden
135) Privatklinik Ritzensee
136) Rehabilitationszentrum Großgmain
137) Emco Privatklinik
138) Klinik St. Barbara - Medizinisches Zentrum Bad Vigaun
139) Bärenhof am Felsenbad
140) PKS Privatklinik Salzburg
141) Krankenanstalt Altenmarkt
142) Krankenhaus Abtenau
143) NeuroCare Rehaklinik Salzburg
144) Rehabilitationszentrum für Stoffwechsel- und Gastroenterologische Erkrankungen
145) Rehabilitstionszentrum Oberndorf
146) Rehabilitationszentrum Bad Vigaun
147) Sonderkrankenanstalt für Neurorehabilitation für Kinder und Jugendliche
148) Landesklinik St Veit (gehört zu K533)
149) Onkologisches Rehabilitationszentrum St. Veit/Pongau
150) Rehabilitationszentrum Aflenz

151) Rehabilitationszentrum Bad Aussee
152) Klinikum Bad Gleichenberg für Lungen- und Stoffwechselerkrankungen
153) Krankenhausverbund Feldbach-Fürstenfeld
154) AMEOS Klinikum Bad Aussee
155) Landeskrankenhaus-Universitätsklinikum Graz
156) Unfallkrankenhaus Steiermark
157) Albert Schweitzer Klinik
158) Militärspital 1, Belgier-Kaserne Graz
159) Privatklinik für psychosomatische Therapie
160) Krankenhaus der Barmherzigen Brüder Graz
161) Krankenhaus der Elisabethinen Graz
162) Privatklinik Leech
163) Privatklinik der Kreuzschwestern
164) Sanatorium St. Leonhard für Frauenheilkunde und Geburtshilfe
165) Lebenswelten der Barmherzigen Brüder - Steiermark
166) Sonderkrankenanstalt der PVA, REHABZ für Erkrankungen des Stütz- und Bewegungsapparates und neurolgische Erkrankungen
167) Landeskrankenhaus Hartberg
168) Neurologisches Therapiezentrum Kapfenberg
169) Landeskrankenhaus Hochsteiermark
170) Landeskrankenhaus Mürzzuschlag
171) Krankenanstaltenverbund Rottenmann-Bad Aussee
172) Rehabilitationszentrum St. Radegund
173) Klinik Diakonissen Schladming
174) Rehabilitationsklinik Tobelbad
175) Marienkrankenhaus Vorau
176) Landeskrankenhaus Südsteiermark
177) Landeskrankenhaus Weiz
178) Hansa Privatklinikum
179) Privatklinik St. Radegund für Innere Medizin, Psychiatrie, Neurologie, Orthopädie und Geriatrie/Gerontologie
180) Landeskrankenhaus Weststeiermark
181) Albert Schweitzer Klinik (gehört zu K615)
182) Privatklinik Kastanienhof
183) Privatklinik Laßnitzhöhe
184) Theresienhof, Klinikum für Orthopädie und orthopädische Rehabilitation
185) Reha Radkersburg, Radkersburger Hof - SKA zur Rehabililitation neurologischer, orthopädischer und rheumatischer Erkrankungen sowie ambulante Untersuchungen und Behandlungen
186) Landeskrankenhaus Murtal
187) LKH Graz II
188) Privatklinik Graz-Ragnitz
189) Sonderkrankenanstalt für orthopädische und neuroorthopädische Rehabilitiation
190) Rehabilitationsklinik für Neurologie, Pädiatrie, Orthopädie und Onkologie
191) Pflegeanstalt für chronisch Kranke
192) Pflegeanstalt für chronisch psychisch Kranke Neutillmitsch-Gralla
193) OptimaMed Rehabilitationszentrum Wildbad
194) Rehabilitationszentrum Bad Häring
195) Landeskrankenhaus Hall in Tirol
196) Rehabilitationszentrum Kitzbühel
197) Landeskrankenhaus Universitätskliniken Innsbruck
198) Ö. Landeskrankenhaus Hochzirl - Natters
199) Sanatorium Kettenbrücke der Barmherzigen Schwestern

200) Privatklinik Hochrum - Sanatorium der Kreuzschwestern Innsbruck
201) Medalp Sportclinic Imst
202) Bezirkskrankenhaus Lienz
203) Bezirkskrankenhaus Reutte
204) Bezirkskrankenhaus St. Johann in Tirol
205) Bezirkskrankenhaus Schwaz
206) Krankenhaus St. Vinzenz Zams
207) heereseigene Sonderkrankenanstalt INNSBRUCK
208) Rehabilitationszentrum Ederhof
209) Bezirkskrankenhaus Kufstein
210) Kursana Sanatorium Wörgl
211) Wittlinger Therapiezentrum - Walchsee
212) Rehabilitationszentrum Münster - Klinikum für Rehabilitation in Tirol für Neurologie, Kardiologie und Pulmologie und Onkologie
213) Landeskrankenhaus Bludenz
214) Landeskrankenhaus Bregenz
215) Krankenhaus der Stadt Dornbirn
216) Landeskrankenhaus Hohenems
217) Landeskrankenhaus Rankweil
218) Pflegeabteilung des LKH Rankweil (gehört zu K824)
219) Krankenhaus Stiftung Maria Ebene Frastanz
220) Landeskrankenhaus Feldkirch
221) Unfallsanatorium Dr. Rhomberg
222) Sanatorium Dr. Schenk
223) Rehabilitationsklinik Montafon
224) Hospiz am See
225) Universitätsklinikum AKH Wien
226) Anton-Proksch-Institut
227) Krankenhaus der Barmherzigen Brüder
228) Krankenhaus der Barmherzigen Schwestern vom heiligen Vinzenz von Paul
229) Privatklinik Confraternität
230) Rehaklinik Baumgarten
231) Evangelisches Krankenhaus
232) Wiener Gesundheitsverbund Klinik Favoriten
233) Privatklinik Goldenes Kreuz
234) Österreichische Gesundheitskasse - Mein Hanusch-Krankenhaus
235) Sanatorium Hera
236) Herz Jesu-Krankenhaus
237) FRANZISKUS SPITAL
238) Wiener Gesundheitsverbund Klinik Hietzing
239) Wiener Gesundheitsverbund Klinik Landstraße
240) St. Josef-Krankenhaus
241) Traumazentrum Wien der Allgemeinen Unfallversicherungsanstalt
242) Wiener Gesundheitsverbund Klinik Ottakring
243) Wiener Gesundheitsverbund Pflege Baumgarten
244) Sonderkrankenanstalt der Justizanstalt Wien-Josefstadt
245) Haus der Barmherzigkeit Seeböckgasse
246) Heereskrankenanstalt Wien
247) Wiener Gesundheitsverbund Pflege Liesing
248) Orthopädisches Spital Speising
249) Rehabilitationszentrum Meidling der Allgemeinen Unfallversicherungsanstalt
250) Rudolfinerhaus
251) Sanatorium Liebhartstal

252) St. Anna-Kinderspital Zentrum für Kinder- und Jugendheilkunde
253) Wiener Privatklinik
254) Krankenhaus Göttlicher Heiland
255) Wiener Gesundheitsverbund Klinik Donaustadt
256) Wiener Gesundheitsverbund Pflege Donaustadt
257) Privatklinik Döbling - Vienna International Health Center
258) Privatklinik Währing
259) Wiener Gesundheitsverbund Klinik Penzing
260) Neurologisches Rehabilitationszentrum Rosenhügel
261) Haus der Barmherzigkeit Tokiostraße
262) Wiener Gesundheitsverbund Klinik Floridsdorf
263) Wiener Gesundheitsverbund Pflege Meidling
264) Wiener Gesundheitsverbund Pflege Leopoldstadt
265) Wiener Gesundheitsverbund Pflege Simmering
266) Wiener Gesundheitsverbund Pflege Rudolfsheim-Fünfhaus
267) Wiener Gesundheitsverbund Pflege Innerfavoriten
268) Kuzbari Zentrum für ästhetische Medizin
